# Supplementary material for: Metagenomic next-generation sequencing to characterize potential etiologies of non-malarial fever in a cohort living in a high malaria burden area of Uganda
Source: PLOS Glob Public Health. 2023 May 3;3(5):e0001675. doi: 10.1371/journal.pgph.0001675 (PMC10156012; doi:10.1371/journal.pgph.0001675)
Supplement: S1 Checklist — (DOCX) [file pgph.0001675.s001.docx]

Inclusivity in global research

PLOS’ policy on inclusivity in global research aims to improve transparency in the reporting of research performed outside of researchers’ own country or community and ensures that PLOS publications reporting global research adhere to high standards for research ethics and authorship. Authors of relevant research articles may be asked to complete the questionnaire below, which outlines ethical, cultural, and scientific considerations specific to inclusivity in global research. This questionnaire may be requested when researchers have travelled to a different country to conduct research, if research uses samples collected in another country, research with Indigenous populations or their lands, or if research is on cultural artefacts. Researchers travelling to another country solely to use laboratory equipment will not normally be required to complete the questionnaire. However, the questionnaire can be requested at the journal’s discretion for any submission – if you have been requested to complete this questionnaire by the PLOS journal you submitted to, please do so.

Please complete the questionnaire below and include this as a Supporting Information file with your manuscript. Note that if your paper is accepted for publication, this checklist will be published with your article in the supporting information files. Please ensure that you reference the checklist in the main body of your manuscript. We suggest adding a subsection ‘Inclusivity in global research’ to your Methods section and adding the following sentence: “Additional information regarding the ethical, cultural, and scientific considerations specific to inclusivity in global research is included in the Supporting Information (SX Checklist)”

The questions have been designed to be applicable to a wide range of study types, and there are subsections for both human subjects research and non-human subjects research. If any of the questions are not relevant to your research please mark them as “N/A” as appropriate.

**Ethical considerations, permits and authorship**

*This section is applicable to all research types.*

Provide details as to who granted permissions and/or consent for the study to take place in the Methods section of your manuscript. This should include the names of **all** ethics boards, governmental organizations, community leaders or other bodies that provided approval for the study. If individuals provided approval refer to these people by their role or title but do not list their name(s).

Reported on page number: 6

The study protocol was reviewed and approved by the Makerere University School of Medicine Research and Ethics Committee, the Uganda National Council of Science and Technology, the University of California, San Francisco, Human Research Protection Program, the London School of Hygiene & Tropical Medicine Ethics Committee, and the Stanford University Research Compliance Office. Written informed consent was obtained for all participants prior to enrolment into the study.

If there were any deviations from the study protocol after approval was obtained please provide details of these changes in the Methods section of your manuscript.
Did this study involve local collaborators that are residents of the country where the research was conducted or members of the community studied? If you do not have any authors from said communities, please provide an explanation for this below.

Reported on page number: N/A

Yes

Everyone listed as an author should meet PLOS’ criteria for authorship and all individuals who meet these criteria should be included in the author byline, rather than the acknowledgements. Authorship criteria is based on the International Committee of Medical Journal Editors (ICMJE) Uniform Requirements for Manuscripts Submitted to Biomedical Journals - for further information please see here: <https://journals.plos.org/plosone/s/authorship>.

**Human subjects research (e.g. health research, medical research, cross-cultural psychology)**

Did you obtain written informed consent from a representative of the local community or region before the research took place? How did you establish who speaks for the community? Details of written informed consent obtained from study participants should be reported separately in the Methods section of your manuscript.

The community engagement plan in the overall PRISM cohort study protocol states as follows:

*At the inception of the study, the team will meet with the local district leadership both technical and political to share study plans. For the last ten years, we have had an existing Community Advisory Board (CAB) comprised of local political, religious, civil society, study participant representatives and technical personnel that hold meetings quarterly to review study progress and share feedback from the community. We will continue engaging the CAB and the local leadership through-out the study period.*

How did members of the local community provide input on the aims of the research investigation, its methodology, and its anticipated outcome(s)?

See response to above.

When engaging with the local community, how did you ensure that the informed consent documents and other materials could be understood by local stakeholders?

The informed consent and assent process in the overall cohort study protocol states as follows:

*Written informed consent and assent forms will be available in all of the local languages prevalent in Tororo and Busia districts (**English, Japadhola, Kiswahili, Lusamya and Luganda). Before beginning the informed consent process, study staff should determine if the participant is able to read and/or write. If the adult, child, or parent/guardian is unable to read, a witness should be identified. The witness must be present during the informed consent discussion. Any adult over the age of 18 years who is able to read and write, and who speaks the language that the informed consent discussion is being conducted in, may serve as an impartial witness. The witness may be a family member or any other person the participant agrees to that is not study staff.*

*Study staff will conduct the informed consent or assent discussion in the appropriate language and a translator will be used if necessary and will include a detailed description of the purpose of the study, study procedures and risks and benefits to participants. Each section of the consent form shall be read to the participant or parent/guardian exactly as it is written either by the study staff or by the translator if the study staff cannot read that language, and then further explained to the participant or parent/guardian if necessary. The translator will also assist with the discussion and assessment of comprehension. In the case that the adult or child is illiterate, he/she will be asked to place a thumb print onto an appropriate space on the consent and an impartial witness, who can read the language used in the informed consent process, will be asked to witness the discussion and sign the consent form. If both a witness and a translator are required for the informed consent process, these should be two separate people.*

Will the findings of the research be made available in an understandable format to stakeholders in the community where the study was conducted (e.g. via a presentation, summary report, copies of publications, etc.)? Please provide details of how this will be achieved.

The dissemination and publication of research findings process in the overall cohort study protocol states as follows:

*The results from this research will be communicated to stakeholders through dissemination meetings and to participants using language-appropriate information sheets. Investigators will present results at relevant conferences and submit manuscript(s) to peer-reviewed journals in accordance with the NIH, UCSF, LSHTM, Stanford University, UNCST, and Makerere University guidelines.*

**Non-human subjects research using specimens/ animals collected as part of the study, or those housed in archival collections. Examples include archaeology, paleontology, botany and zoology.**

Did the permission you obtained from a local authority to perform the study include an agreement on access to outputs and benefit sharing? This may include procedures to enable fair distribution of the benefits and resources arising from the research performed. Please include any details of Prior Informed Consent and Benefit Sharing Agreements obtained. These may be required by field-specific regulations, for example the Convention on Biological Diversity (CBD) and the associated Nagoya Protocol.

If the material used in your study was imported, please A) provide the year it was imported and B) indicate whether permits were obtained to import/export the materials used, C) provide details of any permits obtained. If this information is not available, please indicate this.

If you used archival specimens, please state how the material used in your study was acquired by the institute it is held in and provide details of any permits obtained for the original excavations/ sample collection. If this information is not available, please indicate this.

How was the potential cultural significance of the materials collected in your study to local communities considered in your research design? Were Indigenous peoples and/or local researchers and institutions involved with archaeological excavations / collection of specimens? If so, please provide a description of their involvement.

If your manuscript includes photographs of human remains please indicate whether authors obtained permission from descendants or affiliated cultural communities to do so.
