# Supplementary figures and images for: Metagenomic next-generation sequencing to characterize potential etiologies of non-malarial fever in a cohort living in a high malaria burden area of Uganda
Source: PLOS Glob Public Health. 2023 May 3;3(5):e0001675. doi: 10.1371/journal.pgph.0001675 (PMC10156012; doi:10.1371/journal.pgph.0001675)

A

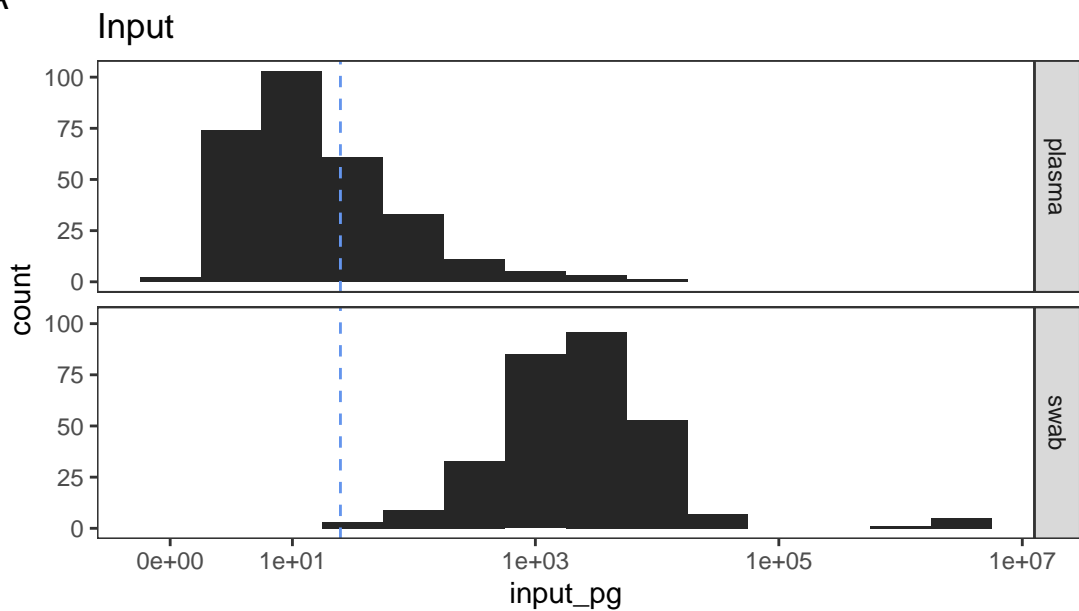

B

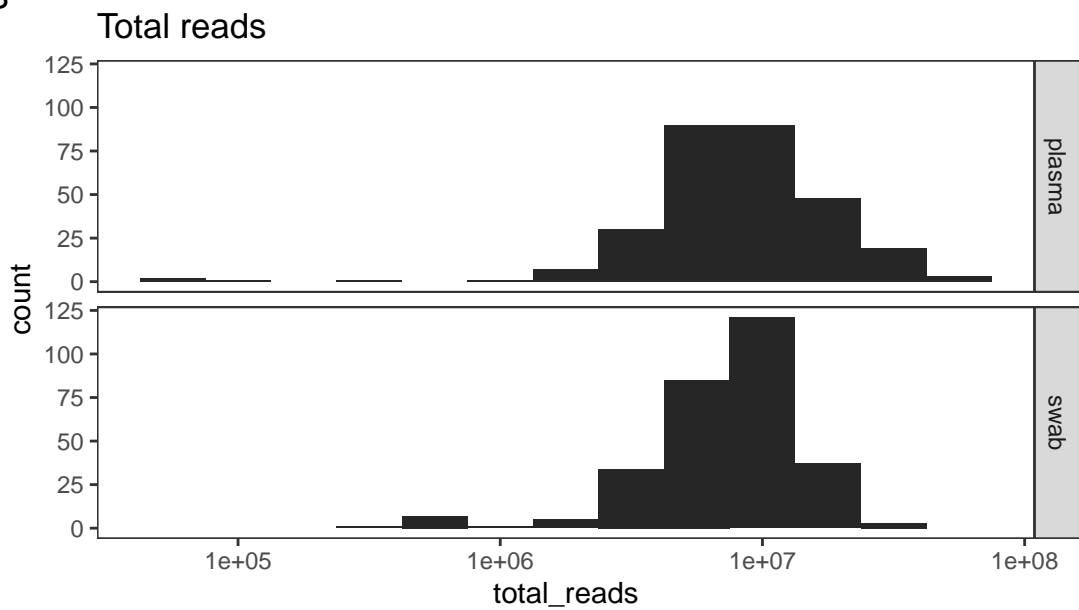

Supplement: S1 Fig — (A) Input RNA in picograms. Blue line indicates 25 pg (i.e., amount of spike-in control in each sample). (B) Total reads per sample. These figures include the 292 plasma samples and 294 swab samples which passed CZ ID’s QC filters and were thus included in this analysis. Sample input is calculated as: (25 pg / ERCC reads) * (total reads—ERCC reads). (PDF) [file pgph.0001675.s002.pdf]

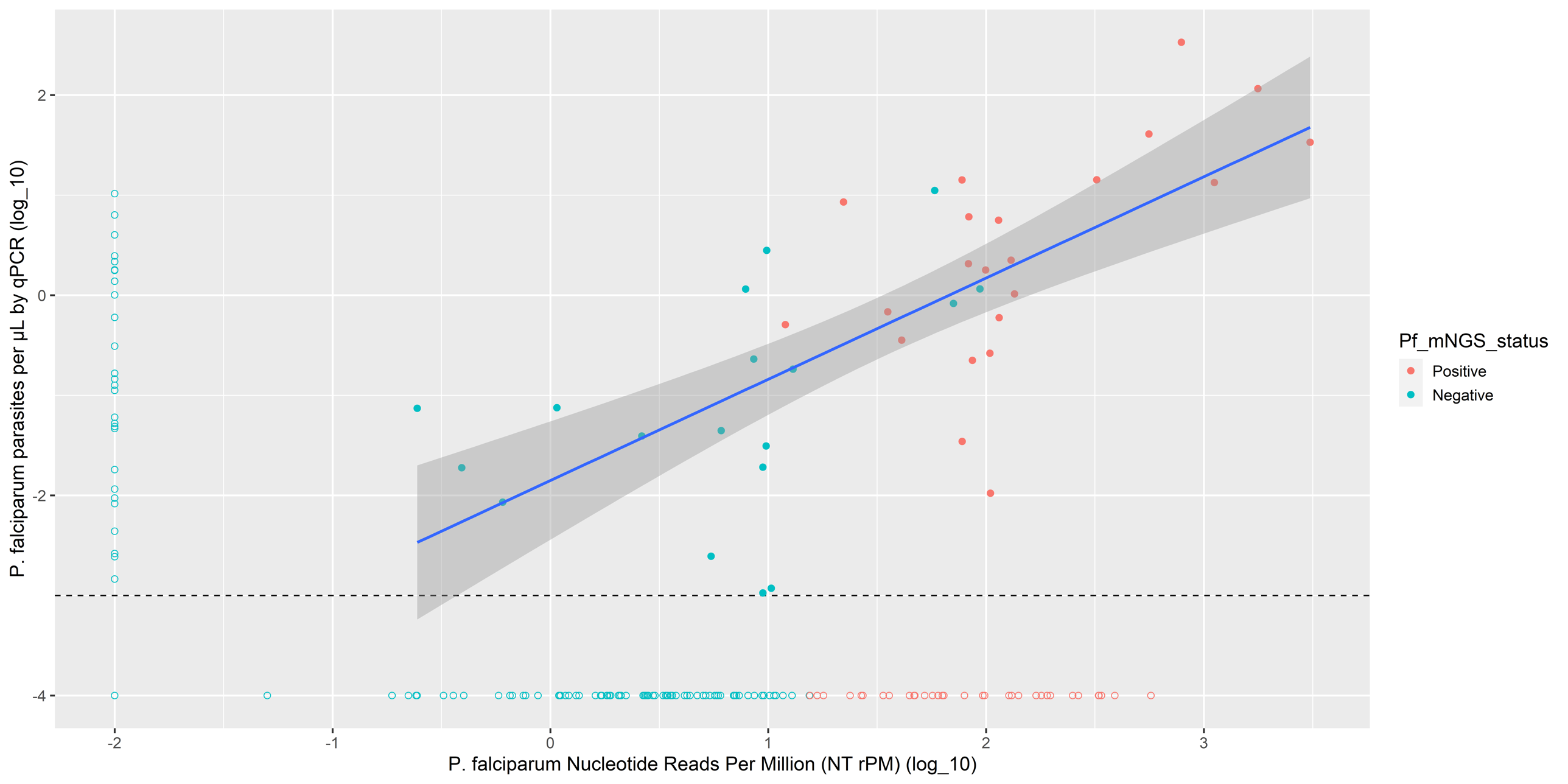

Supplement: S3 Fig — mNGS NT rPMs in plasma samples. The filled points (which have non-zero values for both qPCR and mNGS NT rPM to Plasmodium falciparum) were included in a linear regression of log parasite density (y-axis) versus log NT rPM (x-axis). Note that the model includes samples that did not meet our threshold criteria to be called as positive for Plasmodium falciparum by mNGS (indicated by color). The open points (which have a zero value by either or both assays, and have pseudocounts added for visualization purposes) were excluded from the regression. The horizontal line indicates the threshold to be called positive for Plasmodium falciparum by qPCR. Estimated slope = 1.01 and adjusted R2 = 0.49. (PNG) [file pgph.0001675.s004.png]

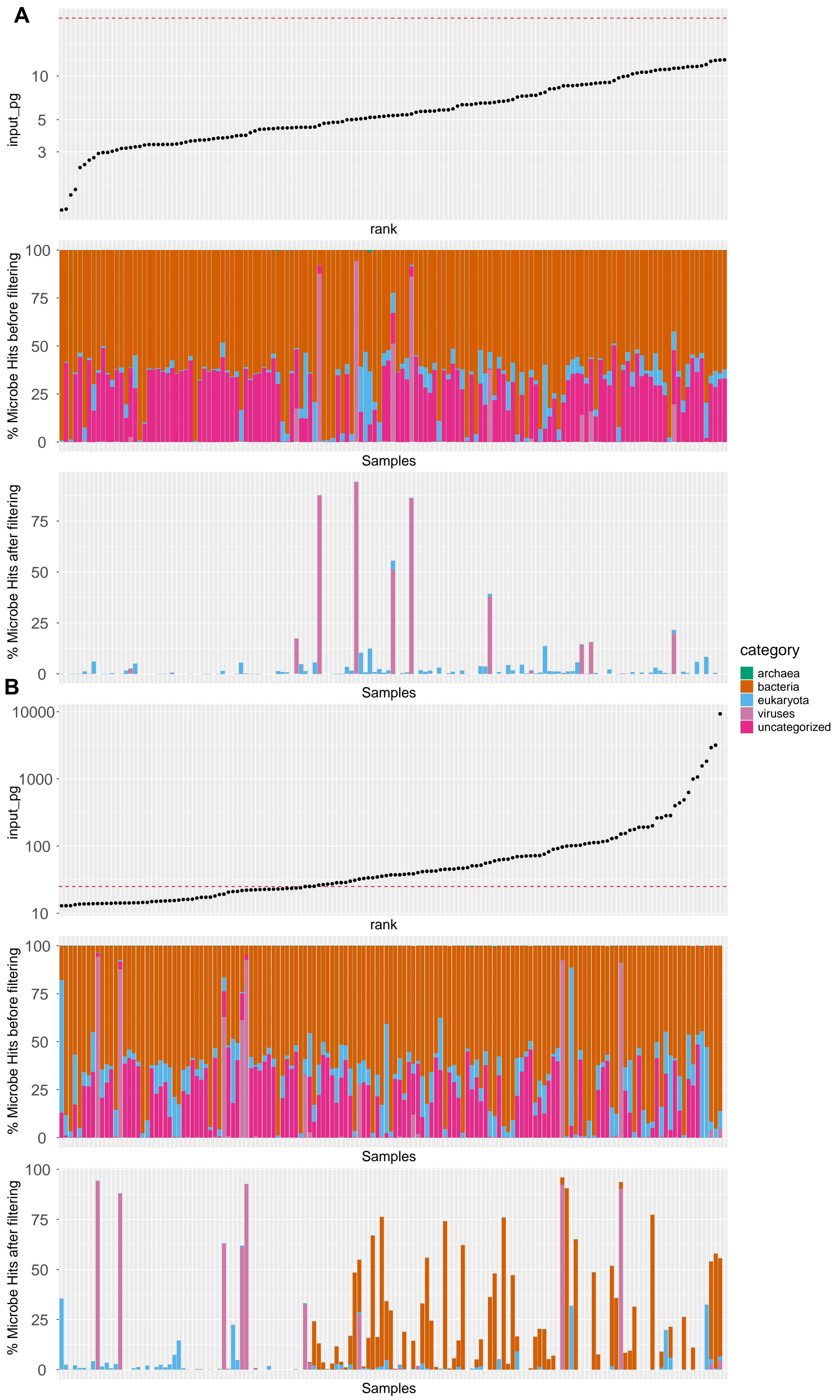

Supplement: S4 Fig — Each column is a distinct sample. The top row shows the sample input. The 25 pg threshold is shown by the dotted red line. The y-axes of this row vary by panel. The middle row represents the proportion of reads within the sample that corresponded to each kingdom prior to filtering. The bottom row represents the proportion of reads within the sample after filtering. Only filtered reads that map to bacterial, viral, or eukaryotic species are included in filtered barplots. Panel (A) includes samples in the bottom half of sample inputs, and panel (B) includes samples in the top half of sample inputs. (PDF) [file pgph.0001675.s005.pdf]

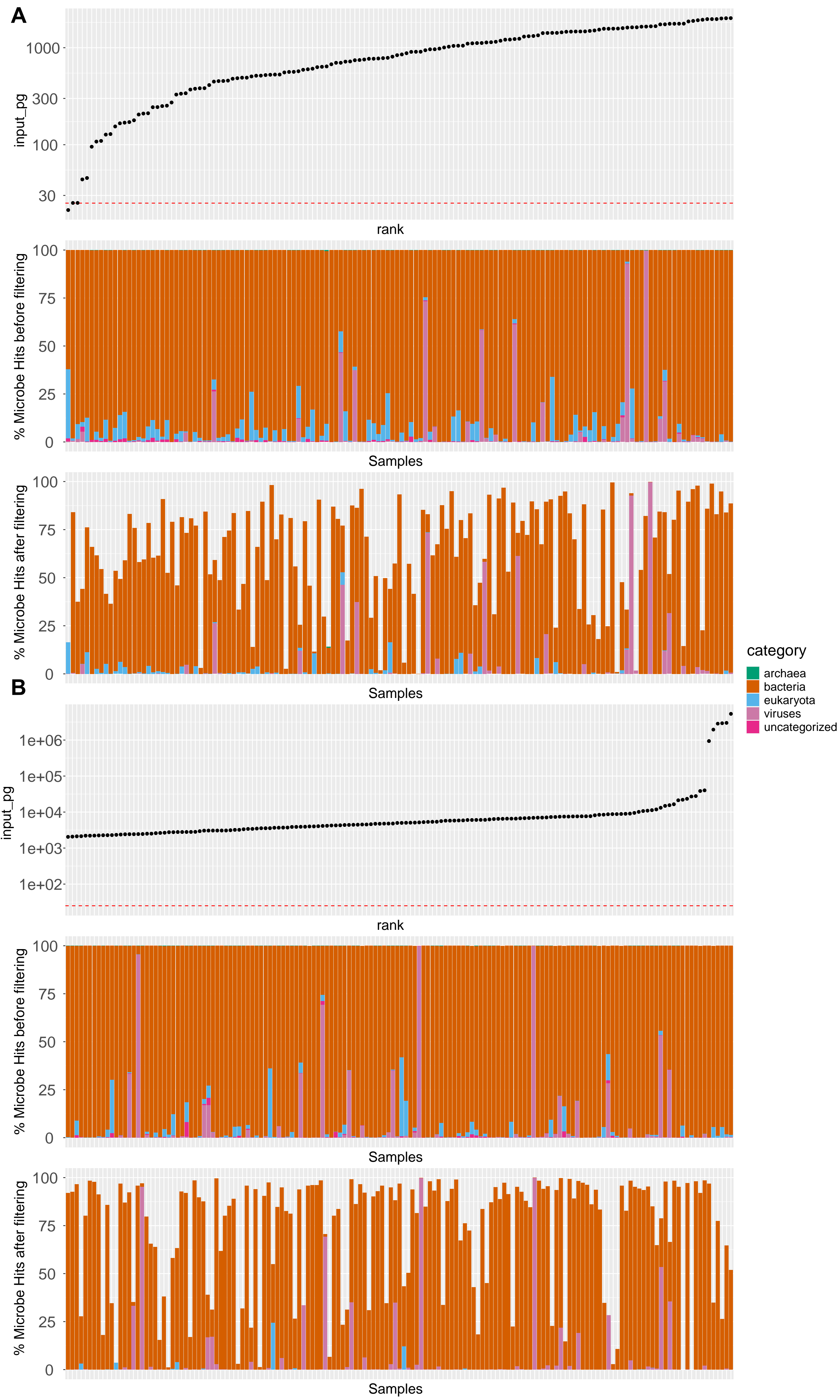

Supplement: S5 Fig — Each column is a distinct sample. The top row shows the sample input. The 25 pg threshold is shown by the dotted red line. The 2 samples in green denote failed ERCC, and thus not the accurate sample input. The y-axes of this row vary by panel. The middle row represents the proportion of reads within the sample that corresponded to each kingdom prior to filtering. The bottom row represents the proportion of reads within the sample after filtering. Only filtered reads that map to bacterial, viral, or eukaryotic species are included in filtered barplots. Panel (A) includes samples in the bottom half of sample inputs, and panel (B) includes samples in the top half of sample inputs. (PDF) [file pgph.0001675.s006.pdf]

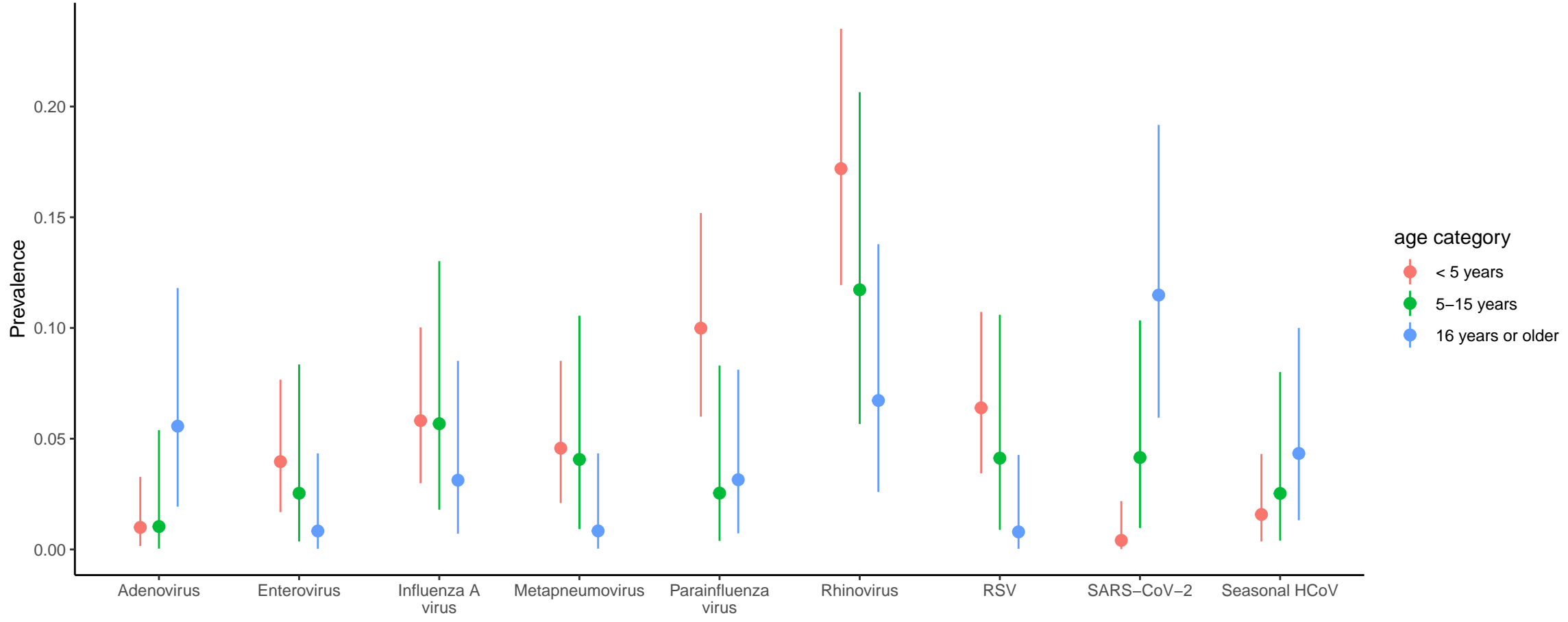

Supplement: S7 Fig — Prevalence was estimated as the probability of detection by mNGS. We assumed that each pathogen detection was independent due to the infrequency of co-detections of respiratory viruses in this study (3 of 119 visits). The point depicts the posterior median probability and the outer interval is the 95% credible interval, using a binomial model. (PDF) [file pgph.0001675.s008.pdf]

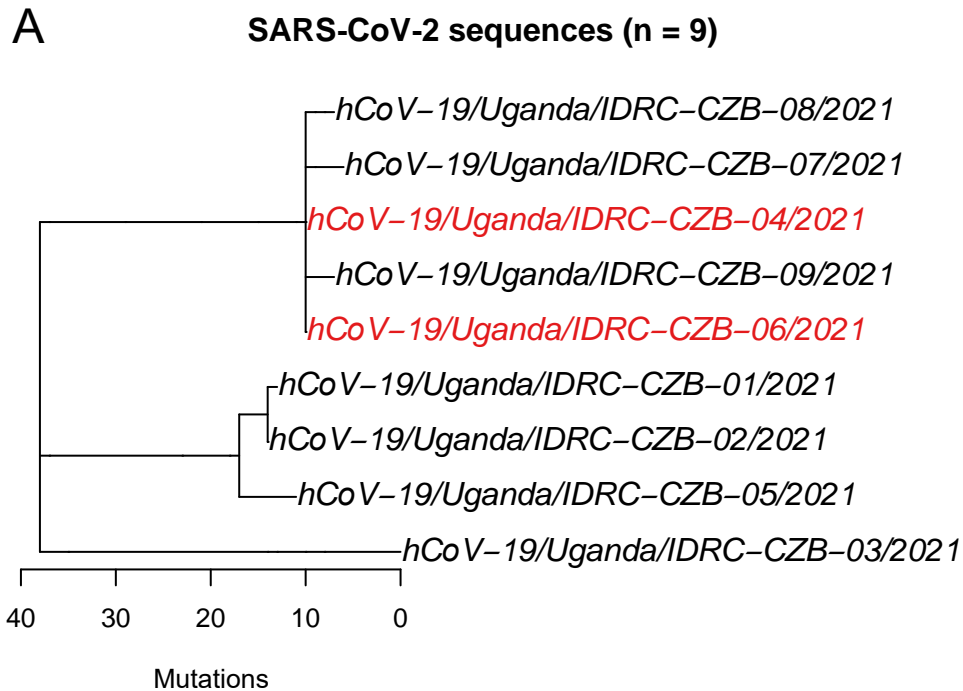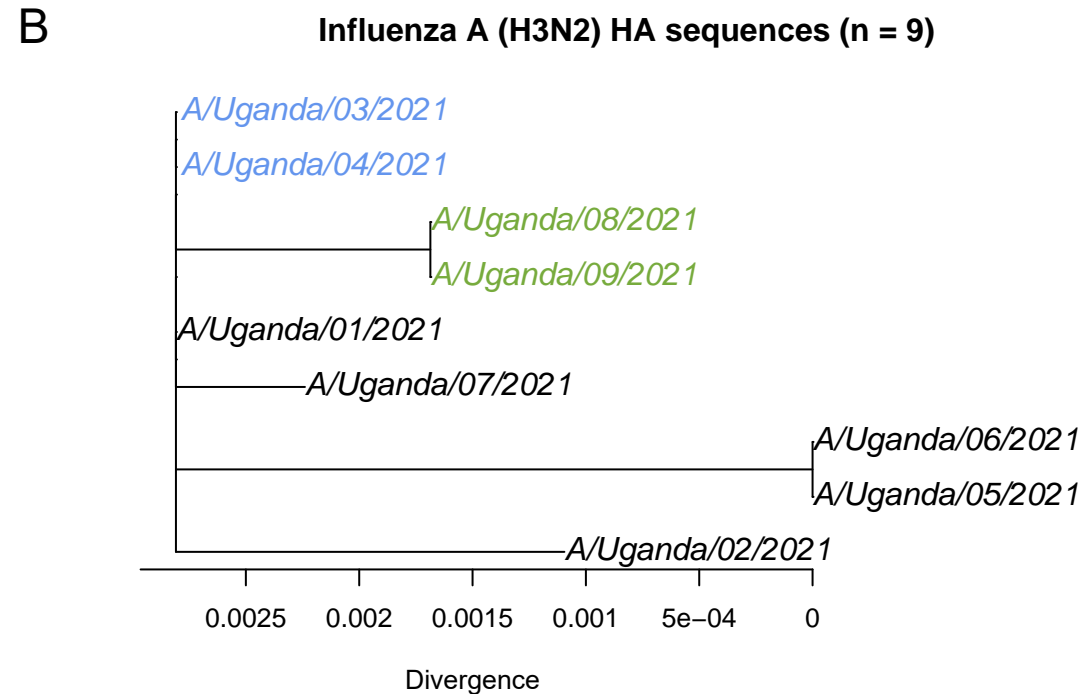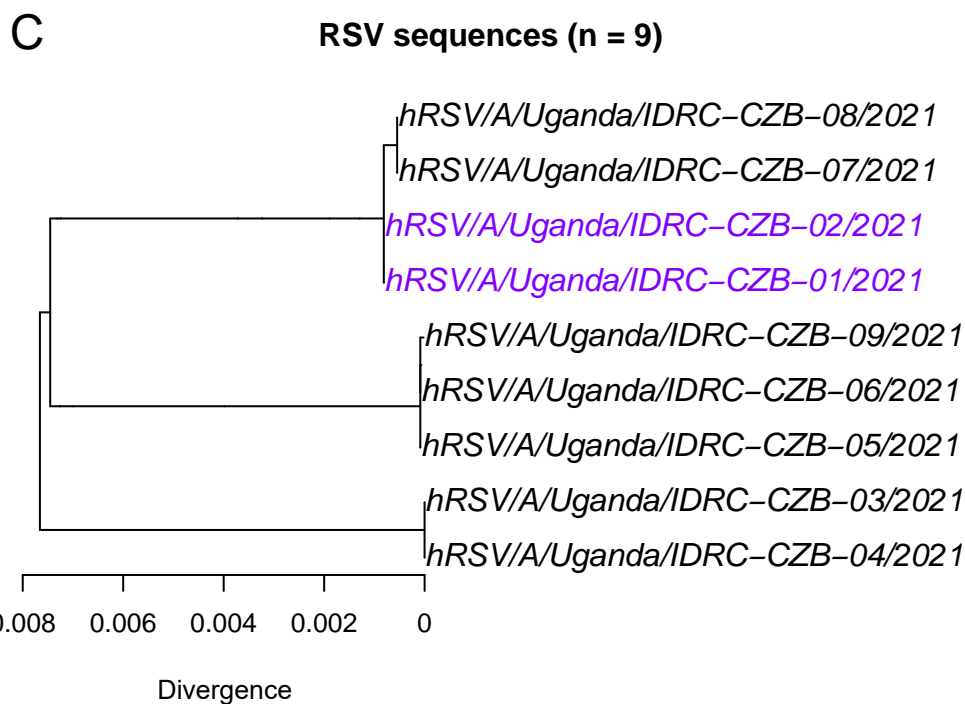

Supplement: S8 Fig — (A) 9 SARS-CoV-2 genomes from this study, with differences in the number of mutations on the x-axis. (B) 9 Influenza A (H3N2) HA gene segments from this study, with divergence (number of mutations per site) on the y-axis. (C) 9 RSV genomes from this study, with divergence (number of mutations per site) on the y-axis. Samples from the same household are shown in the same color (the samples labeled in black are from a household with a singleton sample). (PDF) [file pgph.0001675.s009.pdf]
