# Supplementary material for: Metagenomic next-generation sequencing to characterize potential etiologies of non-malarial fever in a cohort living in a high malaria burden area of Uganda
Source: PLOS Glob Public Health. 2023 May 3;3(5):e0001675. doi: 10.1371/journal.pgph.0001675 (PMC10156012; doi:10.1371/journal.pgph.0001675)
Supplement: S2 Fig — The number of visits with mNGS sample collection among the 212 study participants is provided in the fourth row of Table 1. (PDF) [file pgph.0001675.s003.pdf]

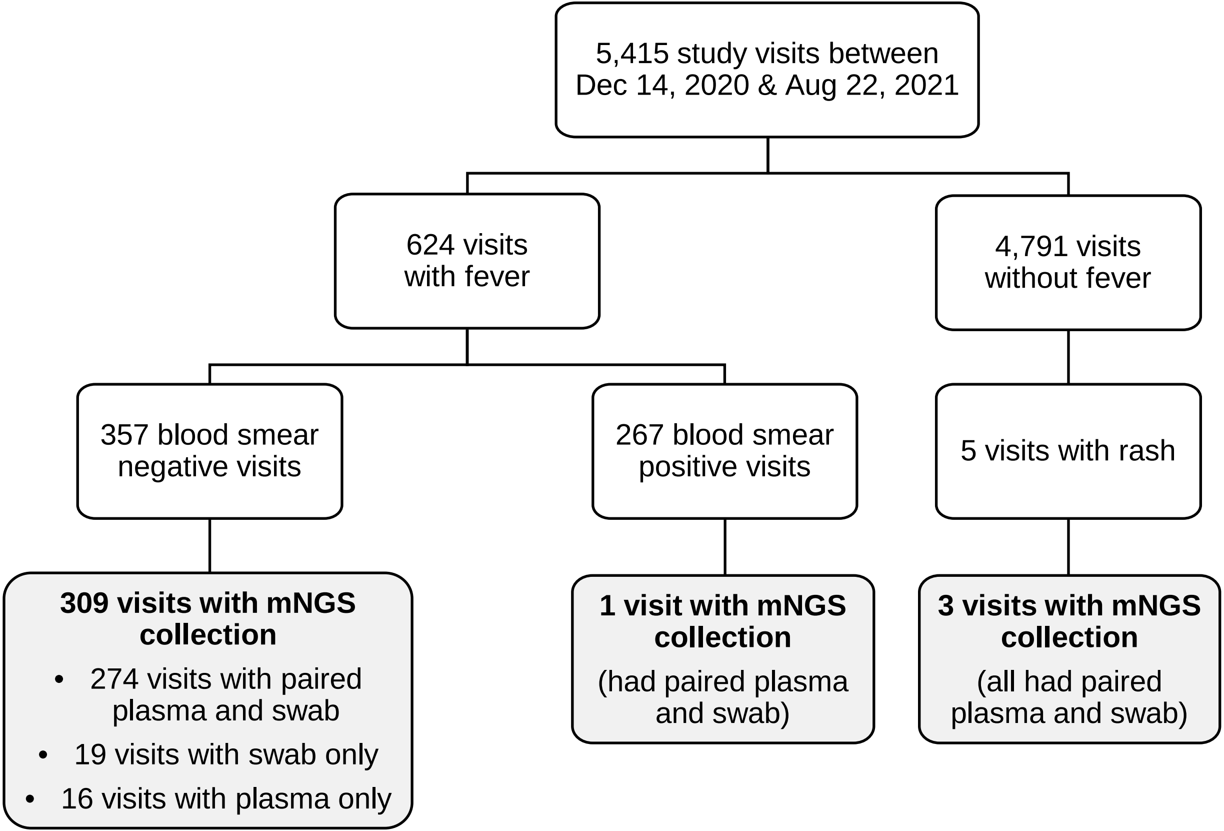
5,415 study visits between Dec 14, 2020 & Aug 22, 2021
624 visits with fever
4,791 visits without fever
357 blood smear negative visits
267 blood smear positive visits
5 visits with rash
309 visits with mNGS collection
• 274 visits with paired plasma and swab
• 19 visits with swab only
• 16 visits with plasma only
1 visit with mNGS collection
(had paired plasma and swab)
3 visits with mNGS collection
(all had paired plasma and swab)
