# Supplementary material for: Metagenomic next-generation sequencing to characterize potential etiologies of non-malarial fever in a cohort living in a high malaria burden area of Uganda
Source: PLOS Glob Public Health. 2023 May 3;3(5):e0001675. doi: 10.1371/journal.pgph.0001675 (PMC10156012; doi:10.1371/journal.pgph.0001675)
Supplement: S6 Fig — All viral microbe species from Fig 2E are included; respiratory and gastrointestinal viral microbes that are known to be pathogenic to humans (see S3 Table) are highlighted with an asterisk. (PDF) [file pgph.0001675.s007.pdf]

Viral detections

In 19 pairs with ≥ 1 viral detection in plasma sample only
(no viral detection in swab)

Pegivirus C (13 detections)
Pegivirus A (10)
Enterovirus A* (2)
Human mastadenovirus C* (2)
Enterovirus B* (1)
Hepatitis GB virus B (1)
Mamastrovirus 1 (1)
Rotavirus A* (1)

In 81 pairs with ≥ 1 viral detection in swab sample only
(no viral detection in plasma)

Rhinovirus C* (17 detections)
Human orthopneumovirus* (10)
Human respirovirus 1* (10)
Influenza A virus* (10)
Human metapneumovirus* (7)
Rhinovirus B* (6)
SARS-CoV-2* (6)
Rhinovirus A* (5)
Human coronavirus OC43* (4)
Human respirovirus 3* (4)
Human bocavirus (1)
Human coronavirus HKU1* (1)
Human coronavirus NL63* (1)
Human orthorubulavirus 2* (1)

In 19 unpaired plasma samples
(no swab collected)

Enterovirus A* (1 detection)
Human orthopneumovirus* (1)
Pegivirus C (1)
Rhinovirus C* (1)

In 21 unpaired swab samples
(no plasma collected)

Influenza A virus* (3 detections)
Human mastadenovirus C* (2)
Human metapneumovirus* (2)
Rhinovirus A* (2)
SARS-CoV-2* (2)
Human respirovirus 1* (1)
Rhinovirus B* (1)
Rhinovirus C* (1)

In 8 pairs with identical viral detection in plasma & swab samples

Pair	Detection in plasma & swab
1	Rhinovirus C*
2	Rhinovirus C*
3	Enterovirus B*
4	Human respirovirus 3*
5	Enterovirus A*
6	Rotavirus A*
7	Rotavirus A*
8	Rhinovirus C*

153 pairs with no viral detection in plasma or swab sample

In 12 pairs with discordant viral detection(s) in plasma & swab samples

Pair	Detection(s) in plasma	Detection(s) in swab
1	Enterovirus A*<br>Human betaherpesvirus 5	Enterovirus A*
2	Human betaherpesvirus 5	Rhinovirus C*
3	Rotavirus A*	Rhinovirus C*<br>Rotavirus A*
4	Human mastadenovirus C*	Human respirovirus 1*<br>SARS-CoV-2*
5	Pegivirus A	Rhinovirus C*
6	Pegivirus A<br>Pegivirus C	SARS-CoV-2*
7	Pegivirus C	Rhinovirus A*
8	Pegivirus C	Human respirovirus 3*
9	Pegivirus A<br>Pegivirus C	Influenza A virus*
10	Pegivirus A<br>Pegivirus C	SARS-CoV-2*
11	Cardiovirus B	Human orthopneumovirus*
12	Norwalk virus*	Human polyomavirus 3
