# Supplementary material for: Metagenomic next-generation sequencing to characterize potential etiologies of non-malarial fever in a cohort living in a high malaria burden area of Uganda
Source: PLOS Glob Public Health. 2023 May 3;3(5):e0001675. doi: 10.1371/journal.pgph.0001675 (PMC10156012; doi:10.1371/journal.pgph.0001675)
Supplement: S1 Table — (PDF) [file pgph.0001675.s010.pdf]

S1 Table: Protocol details for the two processing/sequencing batches.

	Batch 1	Batch 2
**Sample collection dates**	December 14, 2020 to February 6, 2021	February 8, 2021 to August 22, 2021
**Number of swab samples collected (how many passed QC)**	65 (62)	232 (232)
**Number of plasma samples collected (how many passed QC)**	76 (76)	218 (216)
**Number of water controls used to establish background model**	22	84
**Dilution of FastSelect -rRNA HMR for human RNA ribosomal depletion**	1:100	1:10
**Sequencer used**	Illumina NextSeq 2000	Illumina NovaSeq 6000
**Mean paired-end reads per sample**	5.2 million	9.2 million
