# Supplementary material for: Metagenomic next-generation sequencing to characterize potential etiologies of non-malarial fever in a cohort living in a high malaria burden area of Uganda
Source: PLOS Glob Public Health. 2023 May 3;3(5):e0001675. doi: 10.1371/journal.pgph.0001675 (PMC10156012; doi:10.1371/journal.pgph.0001675)
Supplement: S2 Table — (PDF) [file pgph.0001675.s011.pdf]

S2 Table: Accession numbers for SARS-CoV-2, Influenza A virus, and RSV sequences.

**SARS-CoV-2**	
**Virus Name**	**GISAID EpiCoV Accession ID**
hCoV-19/Uganda/IDRC-CZB-01/2021	EPI_ISL_8766868
hCoV-19/Uganda/IDRC-CZB-02/2021	EPI_ISL_8766869
hCoV-19/Uganda/IDRC-CZB-03/2021	EPI_ISL_8766870
hCoV-19/Uganda/IDRC-CZB-04/2021	EPI_ISL_8766871
hCoV-19/Uganda/IDRC-CZB-05/2021	EPI_ISL_8983215
hCoV-19/Uganda/IDRC-CZB-06/2021	EPI_ISL_8766872
hCoV-19/Uganda/IDRC-CZB-07/2021	EPI_ISL_8766873
hCoV-19/Uganda/IDRC-CZB-08/2021	EPI_ISL_8766874
hCoV-19/Uganda/IDRC-CZB-09/2021	EPI_ISL_8766875
**Influenza A**	
**Virus Name**	**GISAID EpiFlu Accession ID**
A/Uganda/01/2021	EPI_ISL_13493341
A/Uganda/02/2021	EPI_ISL_14016105
A/Uganda/03/2021	EPI_ISL_14016106
A/Uganda/04/2021	EPI_ISL_14016107
A/Uganda/05/2021	EPI_ISL_14016108
A/Uganda/06/2021	EPI_ISL_14016109
A/Uganda/07/2021	EPI_ISL_14016110
A/Uganda/08/2021	EPI_ISL_14016111
A/Uganda/09/2021	EPI_ISL_14016112
**RSV**	
**Virus Name**	**GISAID EpiRSV Accession ID**
hRSV/A/Uganda/IDRC-CZB-01/2021	EPI_ISL_14018007
hRSV/A/Uganda/IDRC-CZB-02/2021	EPI_ISL_14018008
hRSV/A/Uganda/IDRC-CZB-03/2021	EPI_ISL_14039044
hRSV/A/Uganda/IDRC-CZB-04/2021	EPI_ISL_14039045
hRSV/A/Uganda/IDRC-CZB-05/2021	EPI_ISL_14039046
hRSV/A/Uganda/IDRC-CZB-06/2021	EPI_ISL_14039047
hRSV/A/Uganda/IDRC-CZB-07/2021	EPI_ISL_14039048
hRSV/A/Uganda/IDRC-CZB-08/2021	EPI_ISL_14039049
hRSV/A/Uganda/IDRC-CZB-09/2021	EPI_ISL_14039050
