# Supplementary material for: Metagenomic next-generation sequencing to characterize potential etiologies of non-malarial fever in a cohort living in a high malaria burden area of Uganda
Source: PLOS Glob Public Health. 2023 May 3;3(5):e0001675. doi: 10.1371/journal.pgph.0001675 (PMC10156012; doi:10.1371/journal.pgph.0001675)
Supplement: S3 Table — (PDF) [file pgph.0001675.s012.pdf]

S3 Table: Classification of viral microbes/pathogens detected.

Viral species name	Other name(s)	Category for this analysis	Known human pathogen?
Cardiovirus B			--
Enterovirus A		Enterovirus	Respiratory
Enterovirus B		Enterovirus	Respiratory
Hepatitis GB virus B			--
Human betaherpesvirus 5			--
Human bocavirus			--
Human coronavirus HKU1	HCoV-HKU1	Seasonal CoV	Respiratory
Human coronavirus NL63	HCoV-NL63	Seasonal CoV	Respiratory
Human coronavirus OC43	HCoV-OC43	Seasonal CoV	Respiratory
Human mastadenovirus C		Adenovirus	Respiratory
Human metapneumovirus		Metapneumovirus	Respiratory
Human orthopneumovirus	Respiratory syncytial virus (RSV)	RSV	Respiratory
Human orthorubulavirus 2	Human parainfluenza virus 2	Parainfluenza virus	Respiratory
Human polyomavirus 3			--
Human respirovirus 1	Human parainfluenza virus 1	Parainfluenza virus	Respiratory
Human respirovirus 3	Human parainfluenza virus 3	Parainfluenza virus	Respiratory
Influenza A virus		Influenza A virus	Respiratory
Mamastrovirus 1			--
Norwalk virus	Norovirus	Norovirus	Gastrointestinal
Pegivirus A			--
Pegivirus C			--
Rhinovirus A		Rhinovirus	Respiratory
Rhinovirus B		Rhinovirus	Respiratory
Rhinovirus C		Rhinovirus	Respiratory
Rotavirus A		Rotavirus	Gastrointestinal
Severe acute respiratory syndrome-related coronavirus	SARS-CoV-2	SARS-CoV-2	Respiratory
