# Supplementary material for: Metagenomic next-generation sequencing to characterize potential etiologies of non-malarial fever in a cohort living in a high malaria burden area of Uganda
Source: PLOS Glob Public Health. 2023 May 3;3(5):e0001675. doi: 10.1371/journal.pgph.0001675 (PMC10156012; doi:10.1371/journal.pgph.0001675)
Supplement: S4 Table — (PDF) [file pgph.0001675.s013.pdf]

S4 Table: Frequency table of binary results for *Plasmodium falciparum* malaria by mNGS and qPCR for the 292 plasma samples tested by mNGS.

**N = 292 visits with plasma samples tested by mNGS**	**Malaria qPCR (-)**<br><br>**N = 224 visits**	**Malaria qPCR (+)**<br><br>**N = 67 visits**	**No malaria qPCR available**<br><br>**N = 1 visit**
**Malaria mNGS (-) in plasma**<br><br>**N = 235 visits**	190 visits	45 visits	0 visits
**Malaria mNGS (+) in plasma**<br><br>**N = 57 visits**	34 visits	22 visits	1 visit
