# Supplementary material for: Metagenomic next-generation sequencing to characterize potential etiologies of non-malarial fever in a cohort living in a high malaria burden area of Uganda
Source: PLOS Glob Public Health. 2023 May 3;3(5):e0001675. doi: 10.1371/journal.pgph.0001675 (PMC10156012; doi:10.1371/journal.pgph.0001675)
Supplement: S5 Table — (PDF) [file pgph.0001675.s014.pdf]

Name of bacterial genus	Number of hits
Acinetobacter	39
Pseudomonas	32
Nocardioides	27
Escherichia	26
Sphingomonas	25
Micrococcus	23
Cutibacterium	20
Deinococcus	17
Phycicoccus	11
Arthrobacter	9
Staphylococcus	9
Delftia	7
Lactobacillus	7
Streptomyces	7
Methylobacterium	6
Prevotella	5
Rhodococcus	5
Veillonella	5
Actinomyces	4
Bacillus	4
Bifidobacterium	4
Clostridium	4
Klebsiella	4
Massilia	4
Neisseria	4
Streptococcus	4
Wolbachia	4
Xanthomonas	4
Anaerococcus	3
Comamonas	3
Herbaspirillum	3
Mycobacterium	3
Nostoc	3
Pantoea	3
Ralstonia	3
Rhizobium	3
Stenotrophomonas	3
Achromobacter	2
Acidovorax	2
Brevibacterium	2
Calothrix	2
Corynebacterium	2
Curtobacterium	2
Enterobacter	2
Enterococcus	2
Faecalibacterium	2
Finegoldia	2
Fusobacterium	2
Gemella	2
Gemmata	2
Hymenobacter	2
Janibacter	2
Kocuria	2
Leptotrichia	2
Mesorhizobium	2
Methylorubrum	2
Microlunatus	2
non-genus-specific reads in family Nocardioidaceae	2
Paenibacillus	2
Peptoniphilus	2
Propionibacterium	2
Salinicoccus	2
Sphingobium	2
Variovorax	2
Weissella	2

Name of bacterial genus	Number of hits
Actinoplanes	1
Agrobacterium	1
Alistipes	1
Alloprevotella	1
Bacteroides	1
Blastococcus	1
Blautia	1
Bradyrhizobium	1
Brevundimonas	1
Burkholderia	1
Campylobacter	1
Candidatus Methylomirabilis	1
Caulobacter	1
Cellulomonas	1
Chroococcidiopsis	1
Citrobacter	1
Collinsella	1
Cupriavidus	1
Cylindrospermum	1
Dietzia	1
Duncaniella	1
Epilithonimonas	1
Exiguobacterium	1
Filifactor	1
Gardnerella	1
Geitlerinema	1
Gordonia	1
Halomonas	1
Hassallia	1
Hydrogenophilus	1
Isoptericola	1
Knoellia	1
Lachnoclostridium	1
Lactiplantibacillus	1
Leifsonia	1
Leptolyngbya	1
Ligilactobacillus	1
Limosilactobacillus	1
Marmoricola	1
Mediterraneibacter	1
Meiothermus	1
Microbacterium	1
Microcoleus	1
Modestobacter	1
Moraxella	1
Muribaculum	1
Nocardia	1
non-genus-specific reads in family Lachnospiraceae	1
Novosphingobium	1
Ornithinimicrobium	1
Paraburkholderia	1
Paracoccus	1
Phocaeicola	1
Planktothrix	1
Porphyromonas	1
Pseudarthrobacter	1
Pseudonocardia	1
Psychrobacter	1
Rickettsia	1
Rothia	1
Scytonema	1
Serinicoccus	1
Serratia	1
Sphingobacterium	1
Sphingopyxis	1
Tetrasphaera	1
Tolypothrix	1
