# Supplementary material for: Metagenomic next-generation sequencing to characterize potential etiologies of non-malarial fever in a cohort living in a high malaria burden area of Uganda
Source: PLOS Glob Public Health. 2023 May 3;3(5):e0001675. doi: 10.1371/journal.pgph.0001675 (PMC10156012; doi:10.1371/journal.pgph.0001675)
Supplement: S6 Table — (PDF) [file pgph.0001675.s015.pdf]

Name of bacterial genus	Number of hits
Corynebacterium	235
Streptococcus	196
Acinetobacter	185
Staphylococcus	183
Pseudomonas	176
Neisseria	155
Haemophilus	154
Dolosigranulum	152
Moraxella	147
Micrococcus	139
Escherichia	126
Nocardioides	111
Deinococcus	108
Cutibacterium	106
Veillonella	96
Prevotella	94
Bacillus	82
Actinomyces	77
Pantoea	69
Arthrobacter	68
Klebsiella	66
Fusobacterium	65
Leptotrichia	64
Bacteroides	63
Streptomyces	59
Clostridium	58
Rhodococcus	58
Phycicoccus	56
Pasteurella	54
Psychrobacter	49
Sphingomonas	49
Lactobacillus	47
Alloprevotella	46
Bifidobacterium	46
Aggregatibacter	42
Anaerococcus	42
Schaalia	38
Vibrio	38
Campylobacter	37
Salmonella	36
non-genus-specific reads in family Lachnospiraceae	35
Actinobacillus	32
Gemella	32
Glaesserella	32
Mannheimia	32
Xanthomonas	32
Aeromonas	31
Kocuria	30
Rothia	29
Mycobacterium	28
Curtobacterium	27
Enterobacter	25
Avibacterium	24
Enterococcus	24
Gemmata	24
Methylobacterium	24
Serratia	24
Halomonas	22
Blautia	21
Exiguobacterium	20
Sphingobacterium	20
Microlunatus	19
Nocardia	19
Peptoniphilus	19
Porphyromonas	19
Suttonella	19
Weissella	19
Paenibacillus	18
Pseudoalteromonas	18
Shewanella	18
Enhydrobacter	17
Faecalibacterium	17
Treponema	17
Finegoldia	16
Rhizobium	16
Actinoplanes	15
Flavobacterium	15
Selenomonas	15
Capnocytophaga	14
Gordonia	14
Leuconostoc	14
Parabacteroides	12
Helcococcus	11
Lachnoclostridium	11
Lysinibacillus	11
Mycoplasma	11
Phocaeicola	11
Priestia	11
Stenotrophomonas	11
Delftia	10
Microbacterium	10
Modestobacter	10
non-genus-specific reads in family Nocardioidaceae	10
Yersinia	10

Name of bacterial genus	Number of hits
Curvibacter	9
Marinobacter	9
Micromonospora	9
Novosphingobium	9
Ornithobacterium	9
Pseudarthrobacter	9
Anaerostipes	8
Enterocloster	8
Hymenobacter	8
Lachnoanaerobaculum	8
Mediterraneibacter	8
Parvimonas	8
Propionibacterium	8
Ruminococcus	8
Tannerella	8
Azospirillum	7
Brachybacterium	7
Bradyrhizobium	7
Gardnerella	7
Granulicatella	7
Nostoc	7
Paracoccus	7
Photobacterium	7
Roseburia	7
Agrobacterium	6
Burkholderia	6
Coprococcus	6
Hungatella	6
Lachnospira	6
Limosilactobacillus	6
Megasphaera	6
Pedobacter	6
Collinsella	5
Erysipelothrix	5
Eubacterium	5
Hungateiclostridium	5
Kingella	5
Lautropia	5
Massilia	5
Mesorhizobium	5
Riemerella	5
Atopobium	4
Barnesiella	4
Butyrivibrio	4
Chryseobacterium	4
Citrobacter	4
Dietzia	4
Janibacter	4
Listeria	4
Methylorubrum	4
Mycobacteroides	4
Nocardiopsis	4
non-genus-specific reads in family Clostridiales Family XIII. Incertae Sedis	4
non-genus-specific reads in family Ruminococcaceae	4
Paraprevotella	4
Salinivibrio	4
Anaerobutyricum	3
Brevundimonas	3
Brucella	3
Cardiobacterium	3
Catonella	3
Cupriavidus	3
Erysipelatoclostridium	3
Gallibacterium	3
Lawsonella	3
Legionella	3
Marinomonas	3
Mycolicibacterium	3
Odoribacter	3
Peptostreptococcus	3
Pontibacter	3
Providencia	3
Saccharibacillus	3
Solobacterium	3
Sphingobium	3
Spirosoma	3
Streptobacillus	3
Tetrasphaera	3
Achromobacter	2
Aeromicrobium	2
Aminipila	2
Butyricimonas	2
Faecalibacillus	2
Herbaspirillum	2
Herbinix	2
Lactococcus	2
Ligilactobacillus	2
Macrococcus	2
Magnetospirillum	2
Mogibacterium	2
non-genus-specific reads in family Erysipelotrichaceae	2
non-genus-specific reads in family Moraxellaceae	2
non-genus-specific reads in family Prevotellaceae	2
Oribacterium	2
Ornithinimicrobium	2
Planktothrix	2
Ralstonia	2
Salinicoccus	2
Tuwongella	2

Name of bacterial genus	Number of hits
Abiotrophia	1
Acetobacter	1
Acidiphilium	1
Acidovorax	1
Actinomycetospora	1
Agreia	1
Alistipes	1
Alkalihalobacillus	1
Alteromonas	1
Alysiella	1
Amycolatopsis	1
Anaerobacillus	1
Anoxybacillus	1
Aquabacterium	1
Arcanobacterium	1
Arsenicicoccus	1
Aurantimonas	1
Aureimonas	1
Bordetella	1
Brevibacillus	1
Brevibacterium	1
Brochothrix	1
Candidatus Nitrosacidococcus	1
Carnobacterium	1
Cellulomonas	1
Cellvibrio	1
Chromobacterium	1
Chroococcidiopsis	1
Clostridioides	1
Comamonas	1
Devosia	1
Dialister	1
Dichelobacter	1
Dyadobacter	1
Dysgonomonas	1
Eikenella	1
Elizabethkingia	1
Empedobacter	1
Erwinia	1
Ewingella	1
Filimonas	1
Flammeovirga	1
Flavonifractor	1
Frigoribacterium	1
Gluconobacter	1
Glutamicibacter	1
Isoptericola	1
Janthinobacterium	1
Jeotgalicoccus	1
Johnsonella	1
Knoellia	1
Komagataeibacter	1
Kribbella	1
Kutzneria	1
Lactiplantibacillus	1
Leclercia	1
Leptospira	1
Luteimonas	1
Lysobacter	1
Mammaliicoccus	1
Megamonas	1
Methylomonas	1
Mobilicoccus	1
Mobiluncus	1
Morganella	1
Muribaculum	1
Negativicoccus	1
non-genus-specific reads in family Muribaculaceae	1
non-genus-specific reads in family Pasteurellaceae	1
non-genus-specific reads in family Rhodospirillaceae	1
Oceanisphaera	1
Oligella	1
Pelosinus	1
Peptoanaerobacter	1
Phascolarctobacterium	1
Phyllobacterium	1
Planococcus	1
Pseudescherichia	1
Pseudobutyrivibrio	1
Pseudonocardia	1
Pseudoxanthomonas	1
Pusillimonas	1
Rahnella	1
Rickettsia	1
Rodentibacter	1
Rubrobacter	1
Shigella	1
Simonsiella	1
Stomatobaculum	1
Sutterella	1
Tatumella	1
Taylorella	1
Thiomonas	1
Ureaplasma	1
Variovorax	1
Virgibacillus	1
