# Supplementary material for: Metagenomic next-generation sequencing to characterize potential etiologies of non-malarial fever in a cohort living in a high malaria burden area of Uganda
Source: PLOS Glob Public Health. 2023 May 3;3(5):e0001675. doi: 10.1371/journal.pgph.0001675 (PMC10156012; doi:10.1371/journal.pgph.0001675)
Supplement: S7 Table — Days between infections in the 11 households that had multiple individuals with the same respiratory viral pathogen detected during the sampling period. HH: household. (PDF) [file pgph.0001675.s016.pdf]

Household	Virus	Days since first infection in HH
HH_1	Influenza A virus	0
HH_1	Influenza A virus	11
HH_2	Influenza A virus	0
HH_2	Influenza A virus	0
HH_3	Influenza A virus	0
HH_3	Influenza A virus	39
HH_4	Parainfluenza virus	0
HH_4	Parainfluenza virus	21
HH_5	Parainfluenza virus	0
HH_5	Parainfluenza virus	10
HH_6	Rhinovirus	0
HH_6	Rhinovirus	0
HH_6	Rhinovirus	0
HH_7	Rhinovirus	0
HH_7	Rhinovirus	0
HH_8	Rhinovirus	0
HH_8	Rhinovirus	84
HH_9	RSV	0
HH_9	RSV	0
HH_10	SARS-CoV-2	0
HH_10	SARS-CoV-2	11
HH_11	SARS-CoV-2	0
HH_11	SARS-CoV-2	6
