# Supplementary material for: Metagenomic next-generation sequencing to characterize potential etiologies of non-malarial fever in a cohort living in a high malaria burden area of Uganda
Source: PLOS Glob Public Health. 2023 May 3;3(5):e0001675. doi: 10.1371/journal.pgph.0001675 (PMC10156012; doi:10.1371/journal.pgph.0001675)
Supplement: S8 Table — These are for the 273 visits that had paired mNGS sample collections, as summarized in Table 3. The included viruses are those that are designated as “respiratory” or “gastrointestinal” pathogens in S3 Table. (PDF) [file pgph.0001675.s017.pdf]

qPCR result (malaria)	mNGS result (malaria; plasma)	Virus(es) identified by mNGS (plasma or swab)	Number of visits
Negative	Negative	Enterovirus A	2
Negative	Negative	Enterovirus B	2
Negative	Negative	Human coronavirus NL63	1
Negative	Negative	Human coronavirus OC43	2
Negative	Negative	Human mastadenovirus C	2
Negative	Negative	Human metapneumovirus	2
Negative	Negative	Human orthopneumovirus	10
Negative	Negative	Human orthorubulavirus 2	1
Negative	Negative	Human respirovirus 1	7
Negative	Negative	Human respirovirus 3	2
Negative	Negative	Human respirovirus 3 + Rhinovirus C	1
Negative	Negative	Influenza A virus	8
Negative	Negative	Norwalk virus	1
Negative	Negative	Rhinovirus A	4
Negative	Negative	Rhinovirus A + Rhinovirus B	1
Negative	Negative	Rhinovirus B	4
Negative	Negative	Rhinovirus C	14
Negative	Negative	Rotavirus A	1
Negative	Negative	SARS-CoV-2	6
Negative	Positive	Human coronavirus OC43	1
Negative	Positive	Human metapneumovirus	4
Negative	Positive	Human orthopneumovirus	1
Negative	Positive	Human respirovirus 1	1
Negative	Positive	Rhinovirus C	4
Negative	Positive	Rotavirus A	2
Negative	Positive	Rotavirus A + Rhinovirus C	1
Negative	Positive	SARS-CoV-2	1
Positive	Negative	Human coronavirus HKU1	1
Positive	Negative	Human coronavirus OC43	1
Positive	Negative	Human mastadenovirus C + SARS-CoV-2 + Human respirovirus 1	1
Positive	Negative	Human metapneumovirus	1
Positive	Negative	Human respirovirus 1	1
Positive	Negative	Human respirovirus 3	2
Positive	Negative	Influenza A virus	2
Positive	Negative	Rhinovirus A	1
Positive	Negative	Rhinovirus C	1
Positive	Negative	SARS-CoV-2	1
Positive	Positive	Enterovirus A	2
Positive	Positive	Human respirovirus 1	1
Positive	Positive	Human respirovirus 3	1
Positive	Positive	Influenza A virus	1
Positive	Positive	Rhinovirus B	1
Positive	Positive	Rhinovirus C	2
