# Supplementary material for: Metagenomic next-generation sequencing to characterize potential etiologies of non-malarial fever in a cohort living in a high malaria burden area of Uganda
Source: PLOS Glob Public Health. 2023 May 3;3(5):e0001675. doi: 10.1371/journal.pgph.0001675 (PMC10156012; doi:10.1371/journal.pgph.0001675)
Supplement: S1 Text — (DOCX) [file pgph.0001675.s018.docx]

# S1 Text: Supplementary Results.

## SARS-CoV-2 phylogenetic analysis (Fig 5)

We performed phylogenetic inference of the 9 full genomes along with other recent SARS-CoV-2 genomes obtained from GISAID from the region and globally. The 9 full genomes represented multiple variant-of-concern and variant-of-interest lineages, including the Delta (5), Eta (3), and Alpha (1) variants. Four of the Delta-lineage viruses fell within a polytomous clade defined by a C10977T mutation. Two were identical (hCoV-19/Uganda/IDRC-CZB-04/2021 and hCoV-19/Uganda/IDRC-CZB-06/2021) and were obtained from individuals who share a household, and a third (hCoV-19/Uganda/IDRC-CZB-09/2021) had an additional 3 unique mutations. The fourth (hCoV-19/Uganda/IDRC-CZB-07/2021) clustered with two other sequences sampled from Uganda, although the most genetically similar virus within this clade had a divergence of 5 nucleotide mutations from hCoV-19/Uganda/IDRC-CZB-07/2021. The fifth Delta-lineage sequence grouped within a different clade of Delta-lineage viruses defined by a G19117T mutation. Amongst the three Eta-lineage viruses, two grouped together (hCoV-19/Uganda/IDRC-CZB-01/2021 and hCoV-19/Uganda/IDRC-CZB-02/2021), sharing C4570A, C13536T, C21811T mutations. These two viruses were separated by a C21846T mutation that was unique to hCoV-19/Uganda/IDRC-CZB-01/2021. The third Eta-lineage virus (hCoV-19/Uganda/IDRC-CZB-05/2021) was six nucleotides diverged from its nearest neighbor in the tree. The one Alpha-lineage virus, hCoV-19/Uganda/IDRC-CZB-03/2021, grouped together with other viruses sampled from Uganda (clade defined by mutations A2563G and G5992A) but was still 10 nucleotides diverged from its nearest neighbor in the tree. The estimated evolutionary rate was 7.74 x 10^-4^ substitutions per site per year.

## Influenza A virus phylogenetic analysis (Fig 6)

All 9 HA sequences generated in this study were grouped together in a clade defined by C1577T. The three most basal viruses within the clade (A/Uganda/01/2021, A/Uganda/03/2021, and A/Uganda/04/2021) were identical to each other (the latter two of which were obtained from individuals who share a household) and to a third virus sampled from Zambia. Within the clade, our sequences clustered into smaller groups as well. Two identical sequences (A/Uganda/08/2021 and A/Uganda/09/2021), obtained from individuals who share a household, were separated from the basal virus by 2 mutations (C641T and C1679T). Another pair of identical sequences (A/Uganda/05/2021 and A/Uganda/06/2021) were separated from the basal virus by 5 mutations (A453C, T206C, A353G, A797G and G862A). Finally, two terminal sequences had 1 and 3 additional unique mutations, respectively, on top of the basal genotype of this clade. The estimated evolutionary rate was 3.92 x 10^-3^ substitutions per site per year.

## RSV phylogenetic analysis (Fig 7)

The 9 RSV sequences generated as part of this study fell into 3 distinct clades. Clade 1 contained 4 of the 9 viruses. One identical pair of sequences on Clade 1 (hRSV/A/Uganda/IDRC-CZB-01/2021 and hRSV/A/Uganda/IDRC-CZB-02/2021) were obtained from individuals who share a household. Another identical pair of sequences on this clade (hRSV/A/Uganda/IDRC-CZB-07/2021 and hRSV/A/Uganda/IDRC-CZB-08/2021) was separated from the basal virus in this clade by 4 additional mutations (C2670T, T7962C, G10144A and C12109T). The inferred date for the most recent common ancestor for Clade 1 was January 2021 (95% CI: December 2020 to January 2021). Clade 1 was most closely related to samples from South Africa but still quite divergent, with 11 nucleotide mutations separating the inferred most recent common ancestor from the most basal Ugandan samples. The Ugandan and South African samples shared a common ancestor that likely circulated in January 2020 (95% Cl: November 2019 to September 2020). Clade 2 contained 3 of the 9 viruses, two of which were identical (hRSV/A/Uganda/IDRC-CZB-05/2021 and hRSV/A/Uganda/IDRC-CZB-06/2021) and a third, hRSV/A/Uganda/IDRC-CZB-09/2021, with an additional C4616T mutation. The inferred date for the most recent common ancestor for Clade 2 was May 2021 (95% CI: April 2021 to June 2021). Clade 3 contained the remaining 2 viruses, hRSV/A/Uganda/IDRC-CZB-03/2021 and hRSV/A/Uganda/IDRC-CZB-04/2021, which were identical. The inferred date for the most recent common ancestor for Clade 3 was February 2021 (95% CI: January 2021 to February 2021). The estimated evolutionary rate was 8.60 x 10^-4^ substitutions per site per year.
