# Supplementary material for: Metagenomic next-generation sequencing to characterize potential etiologies of non-malarial fever in a cohort living in a high malaria burden area of Uganda
Source: PLOS Glob Public Health. 2023 May 3;3(5):e0001675. doi: 10.1371/journal.pgph.0001675 (PMC10156012; doi:10.1371/journal.pgph.0001675)
Supplement: Attachment — Submitted filename: PRISM Manuscript_Reviewers Comments.docx [file pgph.0001675.s020.docx]

**Journal Requirements:**

1. Please include a complete copy of PLOS’ questionnaire on inclusivity in global research in your revised manuscript. Our policy for research in this area aims to improve transparency in the reporting of research performed outside of researchers’ own country or community. The policy applies to researchers who have travelled to a different country to conduct research, research with Indigenous populations or their lands, and research on cultural artefacts. The questionnaire can also be requested at the journal’s discretion for any other submissions, even if these conditions are not met. Please find more information on the policy and a link to download a blank copy of the questionnaire here: https://journals.plos.org/globalpublichealth/s/best-practices-in-research-reporting. Please upload a completed version of your questionnaire as Supporting Information when you resubmit your manuscript.

We have uploaded a completed version of this questionnaire as **S1 Checklist** in the Supporting Information.

2. In the online submission form, you indicated that your data will be submitted to a repository upon acceptance. We strongly recommend all authors deposit their data before acceptance, as the process can be lengthy and hold up publication timelines. Please note that, though access restrictions are acceptable now, your entire data will need to be made freely accessible if your manuscript is accepted for publication. This policy applies to all data except where public deposition would breach compliance with the protocol approved by your research ethics board. If you are unable to adhere to our open data policy, please kindly revise your statement to explain your reasoning and we will seek the editor's input on an exemption. Please be assured that, once you have provided your new statement, the assessment of your exemption will not hold up the peer review process.

The SRA files of non-host reads for each sample, which represent the raw mNGS data in this analysis, have been deposited and are freely accessible at NCBI (PRJNA870959). The link to this published BioProject has been updated in the main text. We have also added the relevant data on the clinical and demographic characteristics of the study participants as **S1 File** in the Supporting Information. In addition, the CZ ID public link to visualize the mNGS sample reports is freely accessible at the link in the main text. Lastly, the SARS-CoV-2, Influenza A, and RSV genomes generated as part of this study have already been uploaded to their respective GISAID databases and are freely accessible (see Accession IDs in Table S2).

**Additional Editor Comments (if provided):**

The approach is interesting, however, the autorhs have to review the discussion and conclusions following the reviewer´s comments.

We have made revisions throughout – particularly to the discussion and conclusions – as suggested by the referees, with key points being how this mNGS assay is not a diagnostic, but rather a research tool, as well as on the solutions that are needed in order to better interpret mNGS results, including the testing of convalescent samples to establish background models and comparisons to gold-standard assays.

**5. Review Comments to the Author**

Please use the space provided to explain your answers to the questions above. You may also include additional comments for the author, including concerns about dual publication, research ethics, or publication ethics. (Please upload your review as an attachment if it exceeds 20,000 characters)

**Reviewer #1:** The authors here present the application of metagenomic next-generation sequencing (mNGS) for broad genomic-level detection of infectious agents to elucidate the potential causes of non-malarial fevers. Based on their data, they identified a viral pathogen in half of the illnesses in young children and in 40% illness overall. The study seems well-designed, however, the authors are advised to compare their observations with other mNGS datasets to evaluate overlapping potential pathogenic species that might not contribute to fever. The authors are also advised to mention the ICD code for fever as applied in this study. Other specific points that are recommended are as follows:

We thank the reviewer for the helpful comments. Regarding the first issue on comparing observations with other mNGS datasets on febrile illness: the study that is most comparable to ours in terms of the same geographic area (Ramesh *et al* 2019 PLoS One, reference 14, also in Tororo District, Uganda) was conducted in an inpatient hospital setting, and only in children. Given these differences in the study populations, we hesitate to directly compare findings as the comparison would be difficult to interpret. We absolutely agree that a comprehensive meta-analysis on the causes of febrile illness (and other clinical presentations) in different settings and populations would allow us to better contextualize the findings of this study. However, we feel that would be a broader investigation that is out of the scope of this work.

Regarding the second issue on the diagnostic criteria for fever: this was determined as either having a fever at the time of visit (defined as objective temperature > 38°C) or having self-reported fever in the past 24 hours; this is now noted within the text of the manuscript under “Study population and design”. No ICD codes were used for this study.

1. Do the authors think that the vaccination status of the cohort might impact the readout?

None of the participants who contributed samples to this study received a SARS-CoV-2 vaccine during the study period. In the broader cohort (i.e., beyond those individuals who contributed samples to this study), a few participants (n=3) received a SARS-CoV-2 vaccine during the study period, while the vast majority (including all children) did not. Furthermore, Uganda does not have an influenza vaccination program, and vaccines are not available for any of the other respiratory viruses detected by mNGS. Importantly, we did not detect any of the viruses that are targeted in Uganda’s routine childhood immunization program (measles, rubella, polio, Hepatitis B).

2. R codes may be shared for data reproducibility through platforms like GitHub as the methodology needs to be provided at the highest possible resolution. The authors may also like to mention if the analysis done in R is part of the snakefile as it is not mentioned in the manuscript.

This study is a descriptive analysis of a single mNGS data set where the bioinformatics analysis was conducted using an established platform, CZ ID. The hit-calling and downstream visualizations were then generated in R, and we now provide the R code used to generate the overall descriptive figure (Figure 2) in GitHub at: <https://github.com/czbiohub/rapid-response-prism/tree/main/Figure%202>. The other key analytical component of this work is the phylogeographic analyses. For these, we provide Snakefiles to produce them in our GitHub directory, and have updated the “Data availability” section of the Methods accordingly.

3. Line 291 (Results), the number of plasma and swab sample don’t match to Figure S1 or Table S4. May please correct.

We apologize for our lack of clarity on this and Comments #4-6 below. To clarify: 297 swab samples and 294 plasma samples were tested in total (as in Line 291). Of these, 294 swab samples and 292 plasma samples passed CZ ID’s QC filters and were thus included in this analysis (as in Figure S1 and Table S4). We have clarified this in the text as follows:

*297 swab samples and 294 plasma samples collected for this study passed CZ ID’s QC filters and were included in this analysis (3 swab samples and 2 plasma specimens were collected but did not pass QC), and most were paired from the same visit.*

4. 212 individuals should be represented in Figure S2 for better clarity of samples collected.

We would prefer to keep this figure as is, since the unit of this figure is study visits and not individuals. The distribution of the number of study visits with mNGS sample collection among the 212 individuals is provided in Table 1. We have added a pointer to this aspect of Table 1 in the figure legend for Figure S2.

5. Line 311 (Results), the text says ‘additional 35 visits’, however, Table 3 says 34, may please correct.

This discrepancy is because one study visit did not have a qPCR result, and we have clarified this in the text.

6. Line 329 (Results), it says 468 bacteria, however, total from Table S5 (132) and Table S6 (289) is 421, please check.

This discrepancy is because we reported at the species level in Line 329 (to correspond to Figure 2), while we reported at the genus level in the respective sample types in Table S5 and Table S6 in order to increase interpretability.

7. Lines 480-481 (Discussion), may please elaborate on the statement of why the authors think that the relative number of cases may be much more similar?

This hypothesis – that the relative number of cases of SARS-CoV-2 and influenza during this time period may be more similar than the public health surveillance systems suggest (which would be differences by several orders of magnitude) – is based on our findings described in Figure 3: we had 11 detections of SARS-CoV-2 and 14 detections of Influenza A virus. We have added these values to this sentence to clarify this point.

8. Data access from <http://czid.org/pub/7pxmXbpFTL> is not working even after several attempts.

We apologize, this may have been an issue with the CZ ID server. We are currently able to open it on our end (on multiple browsers and machines); please try it again and have the Editor inform us if it is still not working.

**Reviewer #2:** Mwakibete et al. applied mNGS for the epidemiological characterization of respiratory and plasma samples from febrile patients without microscopically diagnosed malaria in Uganda. The approach is interesting and educative for readers, however, I do not agree with all interpretations and conclusions of the researchers.

We thank the reviewer for the helpful comments, and have made several clarifications and updates to address the reviewer’s comments about our interpretations and conclusions. One key aspect is on how this mNGS assay is not a diagnostic, but rather a research tool. Another is on the solutions that are needed in order to better interpret mNGS results, including convalescent samples and comparisons to gold-standard assays.

We noted that the reviewer had responded “Partly” to the question of “*1. Does this manuscript meet PLOS Global Public Health’s publication criteria? Is the manuscript technically sound, and do the data support the conclusions? The manuscript must describe methodologically and ethically rigorous research with conclusions that are appropriately drawn based on the data presented.*” We hope that our responses below and the relevant edits to the manuscript have sufficiently addressed the reviewer’s comments on these topics.

1.) In the first paragraph of their introduction, the authors thoroughly explain the phenomenon of semi-immunity after multiple exposition to pathogenic Plasmodium spp. However, they should also stress the difficulty of discriminating clinically apparent infections from harmless colonization in case of the most facultatively pathogenic microorganisms if they are detected at primarily non-sterile sampling sites (like, e.g., many human mucous membranes).

We agree that the inability to discriminate pathogenic bacterial microbes causatively associated with the febrile illness from harmless colonization/contaminants is a key limitation of our work. We had originally included this in our section on caveats in the Discussion section, and have now also added this point upfront in the last section in the Introduction section, as follows:

*Given the difficulty of discriminating bacterial microbes that are pathogenic from those that are commensal or contaminants, for the purposes of this study we focused on the detections of viruses and P. falciparum malaria by mNGS.*

2.) Materials and methods, sub-heading “study population and design”) The data would be better interpretable if the authors presented diagnostic accuracy values (sensitivity, specificity, limit-of-detection) for the applied reference testing approaches like the mentioned “ultrasensitive qPCR for Plasmodium falciparum malaria”. This also applies for potentially undesired side effects on target nucleic acid sequences associated with the depletion of human RNA (page 7, line 149).

First, we have added the limits of quantification and detection of the qPCR assay used, determined from standardized controls, as follows:

*In addition, a sample was collected for subsequent testing via ultrasensitive qPCR for Plasmodium falciparum malaria[24]; the limit of quantification is 0.1 parasites per µL and the limit of detection is 0.01 parasites per µL.*

Second, the FastSelect inhibits about ~98% of all ribosomal RNA of the human host compared to when it is not used. The FastSelect kit specifically targets human, mouse, rat, and other mammalian species (and should not overlap with malaria). We have added the following text to denote the high specificity in its targeting:

*[…] which is known to knockdown ~98% of human ribosomal RNA targeting the cytoplasmic (5S,5.8S,18S,28S) and mitochondrial (12S,16S) rRNAs, and specifically targets human, mouse, rat, and other mammalian species[22].*

3.) The authors should at least comment in the discussion on the diagnostic accuracy and interpretability of CZ ID-based sequence assignments. If the assignments are based on comparisons with deposited sequences at NCBI’s nucleotide, false sequence deposits will most likely influence the reliability of the assignments.

First, we have added emphasis in the Discussion about the important point that this mNGS assay as applied here is not a diagnostic, but rather should be considered a research tool that would need to be validated with PCR or other clinically-approved assays. We agree with the reviewer that testing with well-characterized assays to establish the sensitivity of mNGS to detect a particular pathogen would greatly improve the interpretability of these data; unfortunately these data do not yet exist.

Second, while CZ ID does not account for possible mis-annotation of sequences in the NCBI database, we incorporated threshold filters in both the nucleotide (NT) and non-redundant protein (NR) sequences to minimize false positive calls from mis-alignments or mis-annotations. When using both threshold filters, a mis-annotation false positive would require a sequence to be mis-annotated in both the NT and NR databases. Furthermore, multiple reads mapping to a microbe species leads to increased confidence of being a true hit, and not a false positive from mis-alignments or mis-annotations. In addition, the benchmarking results of CZ ID’s precision/recall on known data sets yielded high AUPR values, indicating the detection of known organisms at higher abundance than any false positives due to mis-alignments or mis-annotations (<https://chanzuckerberg.zendesk.com/hc/en-us/articles/4418861817748-CZ-ID-Pipeline-Update-Making-our-Pipeline-More-Scalable>). We have added these clarifications in the Methods as follows:

*For each sample, significant microbial detections were determined from the unique reads per million (rPM) that mapped to specific microbial taxa, genera, and species. To do so, we applied the following four threshold filters to determine the presence of a microbe in a sample: nucleotide (NT) Z-score ≥ 1 (calculated from the mass-normalized background model created on CZ ID using the water controls), NT rPM ≥ 10 (aiming for a minimum of at least 10 reads of a mapping to a certain microbe per 1 million reads), non-redundant protein (NR) rPM ≥ 5, and average NT alignment ≥ 50 base pairs. We chose to apply both the NT and NR sequence filter to minimize potential false positive detections from mis-alignment or mis-annotation in the NCBI database. Furthermore, the benchmarking results of CZ ID’s precision/recall on known data sets yielded high AUPR values, indicating the detection of known organisms at higher abundance than any false positives due to mis-alignments or mis-annotations (*[*https://chanzuckerberg.zendesk.com/hc/en-us/articles/4418861817748-CZ-ID-Pipeline-Update-Making-our-Pipeline-More-Scalable*](https://chanzuckerberg.zendesk.com/hc/en-us/articles/4418861817748-CZ-ID-Pipeline-Update-Making-our-Pipeline-More-Scalable)*).*

4.) Materials and methods, sub-heading “mNGS analysis using CZ ID”, lines 170-172: The chosen general exclusion of bacterial reads considerably limits the diagnostic value for infections with expected low pathogen loads, for example, in case of bacteremia / sepsis. The authors should discuss how this limitation might be overcome by future mNGS approaches.

We excluded the bacterial reads in samples with low input RNA mass – with “low” determined as being below the amount of spike-in control in each sample (25 pg) – to avoid false positive calls from the potential amplification of background contaminants from reagents and/or the environment. As stated in the response to the reviewer’s Comment #3 above, the sensitivity of mNGS to detect low-pathogen load infections would also need to be benchmarked against gold-standard assays for each pathogen. In addition, based on our experience with this analysis, we are in future studies working on an approach with collection of paired convalescent samples to better account for background and to improve sensitivity.

We agree that low input is a concern, particularly for sepsis, meningitis, or any cases where antibiotics have been utilized prior to sample collection. In these instances, again, mNGS may need to be complemented with additional targeted assays to increase the ability to detect these pathogens: the approaches that others have taken to detect these pathogens involve using mNGS to first characterize the microbial landscape, followed by targeted depletion or enrichment strategies (e.g., amplicon, DASH, FLASH) to diminish more host background and to amplify only known pathogens. By doing these enrichment/depletion methods and capturing more than just a single locus, these approaches would ideally yield high sequencing breadth and depth, increasing confidence on pathogen calls.

5.) Discussion, first paragraph: Similar as already stated in my first comment, I have serious doubt whether the detected respiratory viruses have always been the reasons for the recorded febrile illnesses, considering the high variance of infection-associated symptoms. The authors should discuss this issue more critical. From my point of view, I don’t really see why viral detections should generally be more etiologically relevant than detections of facultatively pathogenic bacteria.

Please see our response to Comment #1 on highlighting our necessarily selective focus on viral detections in this study. We have also re-worded the Discussion to be in line with this: “We were able to detect a viral pathogen” has been altered to “We detected a viral pathogen”. We do not purport that viral detections are more etiologically relevant than facultatively pathogenic bacterial detections; rather, we did not have confidence in our ability to distinguish pathogenic bacteria from commensal/contaminant bacteria under this current study design. As we detail in the third to last paragraph of the Discussion, we have been collecting convalescent samples from participants in order to address this issue (and please see response to next comment).

We agree that particularly for milder respiratory viral infections (e.g., rhinoviruses), they may not be the etiology of the febrile illness. We have softened the language on viruses as “likely causes of a number of illnesses” to be “potential causes of a number of illnesses”, added this as an additional caveat in the Discussion, and have updated the title of the manuscript to qualify “etiologies” as “potential” here as well. Second, mNGS of convalescent samples will likely provide insight into the prevalence of asymptomatic viral detection, which would provide additional ability to discount the etiological relevance of viral species. Lastly, we emphasize that this is an epidemiological/surveillance study focusing on multi-species viral circulation using a research tool in a study population, rather than a clinical diagnostic study. We have highlighted this point in the Discussion.

6.) Discussion, lines 512-513 “background microbiome models”: The authors should discuss the interference of such background subtraction with the detection of infections associated with low pathogen loads. Will there be a solution for this diagnostic dilemma?

We acknowledge that there is a trade-off between specificity (“subtracting out” commensal microbes) and sensitivity (identifying infection with low pathogen loads). As stated in our response to the reviewer’s Comment #4 as well as in the second to last paragraph of the Discussion, testing with well-characterized assays to establish the sensitivity of the mNGS assay would be a key part of resolving this challenge. In addition, orthogonal data such as host transcriptomics, which we did not assess here, could also shed light in discriminating between viral and bacterial infections.

7.) Discussion, lines 526-528: This comment by the authors strongly supports my point two; so, please provide the respective information (as far as it is available).

We have provided the references to the comparisons done against this mNGS assay for SARS-CoV-2 (vs. viral loads by Ct values, in reference 12) and for malaria (vs. parasite density by qPCR, in this manuscript).

8. Discussion/conclusions, lines 530-534: I do not fully agree with this conclusion. From my point of view, the authors’ work clearly shows the obstacles to be overcome to reliably interpret such multiplexed data rather than providing solutions regarding the interpretation of the diagnostic results. Future research will have to focus on approaches of reliable assignment of etiological relevance to the findings of multiplexed diagnostic assessments in order to guide the clinicians’ decisions. Not to forget the influence of Bayes’ theorem in situations of hypothesis-free testing associated with low pretest-probability. Such questions will require proper answers before mNGS can be reliably used for diagnostic purposes in the future.

As stated in our response to the reviewer’s comments above, the premise of this study is one of viral/genomic epidemiologic research. We have removed the reference to “diagnostic” in the sentence identified by the review to maintain the focus on the utility of mNGS to answer epidemiologic research/surveillance questions, in line with this manuscript. We hope that, based on revisions prompted by the reviewer’s previous comments, we have made it clearer that the premise of this work is to use mNGS as a research tool to understand the pathogen landscape in the study population and to reveal the incidence of infections that may be contributing to illness in a population, and not as a clinical diagnostic. We agree that there are multiple obstacles yet to be overcome in interpreting these types of data, and that any findings would need to be validated with clinically-approved tests until a thoroughly-vetted mNGS clinical tool is available.

**Reviewer #3:** This manuscript describes a RNA-based metagenomic next-gen sequencing (mNGS) analysis of approximately 300 plasma and nasal swab samples from a cohort study in Uganda conducted in 2020/2021, with the general aim of elucidating the causal agent of non-malarial fevers in the region. The dataset is thoroughly described, and the analyses appear to be competently performed, with the CZ ID platform doing much of the mNGS analysis. This work will be of broad interest given it addresses the question of how informative metagenomic sequencing can potentially be for clinical diagnosis and multiplexed genomic epidemiology of infectious diseases. Most importantly, though this manuscript very responsibly addresses some limitations of the study, it does not provide a map for what would be required to use such data to infer causality-how much data would be required, from how many subjects, and what sample types, and in what range of locations, to build some kind of general causal classifier of infectious agents from metagenomic sequencing data? Does this study suggest that is a realistic endeavor?

We thank the reviewer for the helpful comments. To potentially use mNGS data for causal inference, we feel that advancements in at least two key areas are needed. First, this would require an improved understanding of the background of bacterial microbes. Based on our experience with this analysis, we are, in future studies, working on an approach with collection of paired convalescent samples to better account for background and to improve sensitivity. The other important advancement would be dedicated studies comparing mNGS results against gold-standard diagnostic assays for multiple potential pathogens of interest. We have underscored these key next steps in the Discussion section.

The manuscript could be improved by addressing/clarifying the following generally minor issues:

1) Line 80/81: The statement is made that RNA mNGS can quantitatively capture the relative abundance of different microbes/viruses in a clinical sample, and do so in a way that is superior to genomic DNA NGS metagenomic sequencing. There is no citation for this assertion and no validation of this statement in the results presented. I would imagine it could be tricky to evaluate relative abundance of various bacteria/eukaryotes with any precision without knowing more about the baseline transcriptional profiles of many organisms, library biases, etc. Perhaps restrict this statement to RNA viruses, or otherwise cite publications validating this.

We understand that both RNA and DNA mNGS techniques have their advantages and disadvantages. The key advantages of using RNA is that the abundance of RNA is more helpful than DNA in distinguishing whether an infection is active or latent, and that it can detect RNA viruses, which would be missed by DNA libraries. Additionally, RNA libraries have higher sensitivity for microbes with smaller genomes compared to genomic DNA, particularly with the included host RNA depletion step. Conversely, DNA mNGS should be employed for detection of latent viruses, bacterial infections treated with antibiotics to ascertain antimicrobial resistance (AMR), and when samples have poor RNA quality for detection of microbes. We have updated the Methods text to address this as follows:

*There are various considerations for selecting the optimal nucleic acid to evaluate in metagenomic studies[17,18]. RNA libraries are required for capturing RNA viruses, and there is a substantially higher signal than DNA from actively replicating microbes due to the amplification of microbial RNA, especially when combined with host RNA depletion methods. Likewise, selecting DNA for a metagenomic study is important when the RNA quality is low, when identifying latent viruses, or when antibiotics have been administered, which would prevent detection of actively replicating bacteria.*

2) Line 151: The sequencing approach is described generally, but it would be useful here to mention the target coverage (profiled in a supp fig, but most will not dig that up), along with a justification for a certain coverage. Coverage level (and cost) is a major factor in the consideration of scaling up approaches such as this, so it could be useful to address cost vs. information content as an issue in the Discussion.

We have added a line in the Methods section specifying the target coverage of at least 5 million reads per sample, to attain sufficient coverage sequencing depth. Additionally, as specified in the Methods: to increase confidence in a microbe being detected, a minimum of at least 10 reads of a mapping to a certain microbe per 1 million reads was required to call a microbe (as well as other threshold filters being met).

Regarding cost vs. information content: while the specific costs will vary, broadly speaking, there is a trade-off between sensitivity and cost, and these trade-offs will continue to evolve as molecular and informatic techniques improve. While sequencing is still more expensive than some other assays, the cost has been coming down consistently over the last decade, and will likely continue to decrease as it becomes more broadly used. Furthermore, the cost per sample decreases significantly when samples are multiplexed into library pools. We have added in the Discussion as follows:

*Cost is an important factor in the consideration of scaling up approaches such as this; there is a trade-off between sensitivity and cost, and these trade-offs will continue to evolve as molecular and informatic techniques improve.*

3) Line 288: The authors note that 'a quarter' of subjects who were smear-negative for malaria at the time of sample collection were smear-positive at a subsequent visit. That seems like a lot, especially if qPCR was also missing some malaria infections. More precision here would be useful here in citing sample numbers; were subjects that subsequently became smear positive more likely to show Plasmodium reads in the mNGS analysis, even if negative by qPCR? This is the kind of issue that seems important to address if the community is ever going make mNGS data more interpretable with regard to causality.

We apologize for the lack of clarity on this. At every study visit, malaria testing is done in two ways: by microscopic examination of a thick blood smear (a less sensitive assay with results available quickly) and by qPCR (a more sensitive assay that takes longer to obtain results). By design, all 313 study visits included in this analysis were microscopy negative for malaria. At a quarter of these visits (74/313 = 23.6%), participants tested positive for malaria by qPCR. We have re-phrased this sentence for increased clarity as follows:

*Even though participants had to be parasite negative by blood smear to be eligible for this mNGS study and have a sample collected, at a quarter of visits (74/313), participants were subsequently found to be positive for Plasmodium falciparum by ultrasensitive qPCR.*

4) Line 335: What does it mean when swab samples detect zero microbial species? Is that a technical assay failure?

We apologize for the confusion – this was not due to a technical assay failure, but rather that these two swab samples did not pass the threshold filter for NR reads. We have rephrased the sentence to clarify this as following:

*2 swab samples had zero microbes detected at the species level after applying threshold filters.*

5) Line 453: Detection of viral pathogens in clinical samples is key, but the authors should remind readers here or elsewhere in the Discussion that the ultimate goal would be to attribute symptoms to a particular detected pathogen. What would be required for that? Certainly lots of metagenomic profiles from healthy, asymptomatic individuals of various ages, as a start.

We have detailed our thoughts on this question in our responses to Comments #5 and #6 from Reviewer 2 above. To summarize, this mNGS assay is a research tool rather than a diagnostic, and our focus is on characterizing the pathogen landscape in the study population. Attributing symptoms to a particular pathogen would require following up with studies using a more quantitative/sensitive approach such as qPCR on a larger sample set for specific pathogens of interest. To potentially use mNGS data for causal inference, we feel that advancements in at least two key areas are needed. First, this would require an improved understanding of the background of bacterial microbes – the inability to discriminate pathogenic bacterial microbes causatively associated with the febrile illness from harmless colonization/contaminants is a key limitation of our work. Based on our experience with this analysis, we are in future studies working on an approach with collection of paired convalescent samples to better account for background and to improve sensitivity. The other important advancement would be dedicated studies comparing mNGS results against gold-standard diagnostic assays for multiple potential pathogens of interest. Additional data providing evidence for causality, such as including host transcriptomics, could also improve interpretability of mNGS results.
